# A chill brain-music interface for enhancing music chills with personalized playlists

**DOI:** 10.1016/j.isci.2025.114508

**Published:** 2025-12-19

**Authors:** Sotaro Kondoh, Takahide Etani, Yuna Sakakibara, Yasushi Naruse, Yasuhiko Imamura, Takuya Ibaraki, Shinya Fujii

**Affiliations:** 1 Graduate School of Media and Governance, Keio University, Fujisawa, Kanagawa, Japan; 2 Faculty of Environment and Information Studies, Keio University, Fujisawa, Kanagawa, Japan; 3 Keio University Research Center for Music Science, Keio University Global Research Institute (KGRI), Tokyo, Japan; 4 Japan Society for the Promotion of Science, Tokyo, Japan; 5 VIE, Inc., Kamakura, Kanagawa, Japan; 6 Keio University Hospital, Tokyo, Japan; 7 Keio Research Institute at SFC, Fujisawa, Kanagawa, Japan

**Keywords:** cognitive neuroscience, psychology

## Abstract

Music chills are physical sensations, such as goosebumps, linked to intense pleasure and engage the brain’s reward system. However, individual differences in music preferences and neural responses make it difficult to enhance these experiences consistently. To address this issue, we developed the chill brain-music interface (C-BMI), a neurofeedback system that uses an in-ear electroencephalogram (EEG) to create personalized playlists. For each participant, we built two regression models: one predicting pleasure from acoustic features, and another decoding pleasure from EEG data. Using these models, four playlists were generated: two designed to enhance pleasure and two to reduce it. In each pair, one playlist incorporated real-time EEG-based updates, whereas the other relied solely on acoustic features. The EEG-updated pleasure-enhancing playlist elicited more subjective chills and higher pleasure ratings than the pleasure-reducing playlists, suggesting that adapting music selection to individual neural activities can amplify chills and emotional engagement with music.

## Highlights

• Musical pleasure was decoded from an in-ear electroencephalogram
• A prediction model of pleasure was updated using the EEG and acoustic data
• The model ranked songs by predicted pleasure, and a song was recommended
• A playlist of top-ranked songs evoked more music chills and higher pleasure

## Graphical abstract

**Figure:**
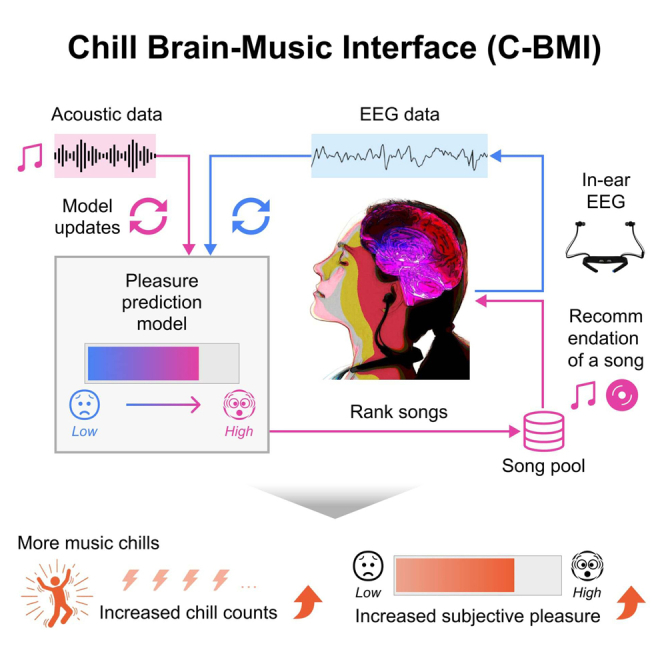

## Introduction

Humans enjoy music universally across diverse cultures and societies.^1^^,^^2^ Although the evolutionary functions of music remain controversial,^3^^,^^4^^,^^5^ it is widely acknowledged that music can evoke positive emotions accompanied by bodily sensations.^6^^,^^7^^,^^8^^,^^9^^,^^10^^,^^11^ One prominent physiological marker is the experience of music chills, which are sensations such as “goosebumps” or a “shiver down the spine” that often accompany intense musical pleasure.^12^ Music is an effective stimulus for eliciting chills,^13^ and these sensations are commonly reported.^14^ A growing body of research has examined the psychological and physiological aspects of music chills,^15^ with many studies showing that chills are accompanied by sympathetic nervous system activity, including increased skin conductance,^16^^,^^17^^,^^18^^,^^19^^,^^20^^,^^21^ elevated heart rate,^16^^,^^17^^,^^19^^,^^20^ piloerection,^20^ and pupil dilation,^22^ suggesting a strong association with emotional arousal.

Neuroimaging studies have demonstrated that experiencing music chills activates the brain’s reward system. For example, music chills increase blood flow to areas including the ventral striatum, anterior cingulate cortex, and insula, which are also activated by rewarding stimuli, such as food, sex, and drugs.^23^ Further research indicates that the dorsal and ventral striatum (i.e., the caudate nucleus and nucleus accumbens) are involved in anticipating and experiencing music chills, respectively.^24^ Moreover, when participants perceived the reward value of the music they listened to for the first time, the functional connectivity between the nucleus accumbens and the auditory cortex strengthened.^25^ These studies indicate that music chills involve both the reward and auditory perceptual systems.^7^ Such pleasurable experiences may reflect positive reward prediction errors that arise when the perceived sound is better than expected.^7^^,^^26^^,^^27^^,^^28^^,^^29^

Researchers have attempted to enhance music chills by stimulating the brain’s reward system. For instance, excitatory intermittent theta-burst transcranial magnetic stimulation of the left dorsolateral prefrontal cortex elicits more pleasure than inhibitory continuous theta bursts.^30^ Taking a dopamine agonist as a pharmacological approach increased subjective music chills and pleasure more than a dopamine antagonist did, without a significant difference in the skin conductance.^31^ Meanwhile, an opioid agonist increases skin conductance without modulating subjective chills,^32^ suggesting that dopamine regulates subjective responses, whereas opioids regulate physiological responses to music rewards. However, individual differences in musical preferences and sensitivity to musical rewards present a challenge for standardized methods to enhance chills. Many previous studies have used experimenter-selected music,^15^ which may be insufficient for examining individual differences in the experience of chills. However, using participant-selected music can address the issues of individual variation. For example, in the study by Grewe et al.,^18^ the number of chills was much higher for participant-selected than for experimenter-selected music. In addition, individuals prone to experiencing music chills show stronger structural connectivity between the superior temporal gyrus, which is involved in auditory processing, and the anterior insula and medial prefrontal cortex, which are associated with emotion and social cognition, than those who are less prone to chills.^33^ Moreover, functional connectivity between the auditory cortex and reward-related areas suggests various musical preferences, as individuals have different auditory templates based on their music exposure and cultural or social influences.^7^^,^^25^ The Barcelona Music Reward Questionnaire (BMRQ) reveals individual variability in musical reward sensitivity and its neural substrates.^34^^,^^35^^,^^36^^,^^37^ Thus, developing methods to improve music chills requires more information about individuals’ musical preferences and their neural responses to musical pleasure.

One promising approach is closed-loop neuromodulation, which adjusts stimuli in real time based on neural activity to guide the brain toward the desired state. Because the system dynamically tailors stimulation to an individual’s neural response, it offers an effective strategy for addressing inter-individual variability. In a notable clinical example, electrodes were implanted in a patient with treatment-resistant depression to deliver targeted stimulation based on real-time neural signals, resulting in symptom relief.^38^ Although this method involves invasive brain stimulation, it illustrates the potential of personalized interventions based on real-time neural monitoring. In contrast, music is a powerful noninvasive modality for closed-loop neuromodulation. Previous studies have demonstrated that electroencephalogram (EEG) signals reflect neural activity associated with music-induced pleasure.^39^^,^^40^^,^^41^^,^^42^^,^^43^^,^^44^ Notably, even individuals who did not initially enjoy music showed increased enjoyment and reduced symptoms of depression and anxiety when neural markers of pleasure were enhanced by sound modulation.^44^ EEG signals thus represent a promising tool for enabling the real-time closed-loop modulation of music.

The present study investigated the effectiveness of the “Chill Brain-Music Interface (C-BMI),” a closed-loop song selection system individually optimized using in-ear EEG activity to induce music chills. We hypothesized that an in-ear EEG-based pleasure-enhancing playlist would elicit more chills than other playlists. Indeed, a playlist that adapted song selection to in-ear EEG signals while considering individual acoustic preferences induced more chills and higher pleasure ratings than pleasure-diminishing playlists. These findings suggest that integrating musical preferences with neural markers of reward processing offers a practical approach for enhancing chills.

## Results

### Overview of the chill brain-music interface

**Figure 1:**
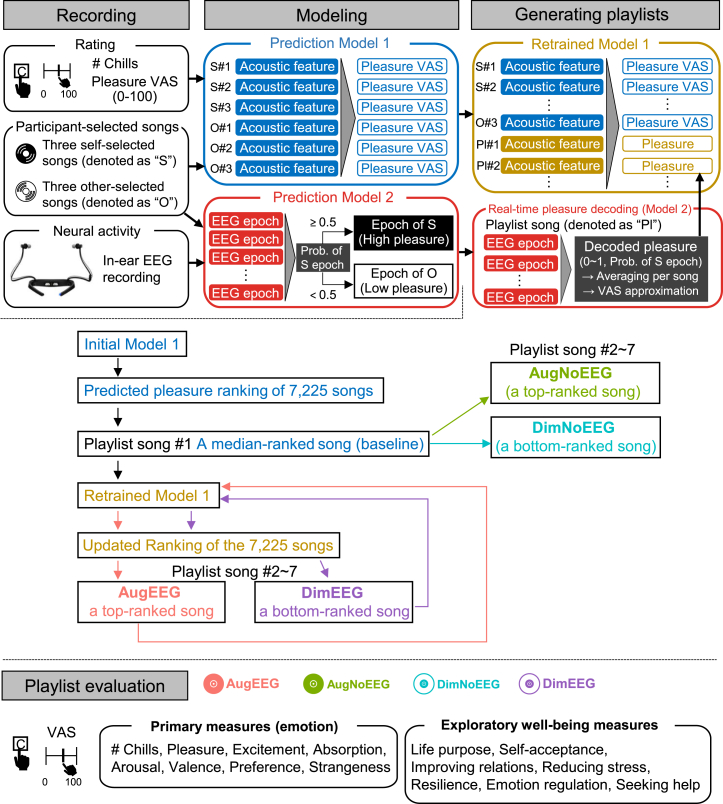
Overview of the “Chill Brain-Music Interface (C-BMI)” In the “Recording” phase, participants listened to three self-selected songs and three songs selected by another participant. They pressed a key whenever they experienced music chills and rated the pleasure level of each song using a visual analog scale (VAS) after listening. The in-ear EEG was recorded simultaneously. In the “Modeling” phase, we developed two prediction models. Model 1 predicted subjective pleasure based on the acoustic features of the participant-selected songs. Model 2 classified EEG activity recorded during self-selected (high pleasure) and other-selected (low pleasure) songs, enabling real-time pleasure decoding from in-ear EEG. In the “Generating playlists” phase, we generated four personalized playlists: AugEEG (augmenting playlist with EEG), DimEEG (diminishing playlist with EEG), AugNoEEG (augmenting playlist without EEG), and DimNoEEG (diminishing playlist without EEG). Model 1 predicted the pleasure level of 7,225 candidate songs, which were then ranked based on the predicted pleasure and acoustic similarity to the self-selected songs. Songs for the augmenting playlists were selected from the top of the ranking, whereas those for the diminishing playlists were selected from the bottom of the ranking. In AugEEG and DimEEG, Model 2 decoded real-time pleasure levels from EEG while participants listened to the songs, and Model 1 was retrained using the decoded pleasure values and acoustic features of the selected songs to update the song rankings. In contrast, AugNoEEG and DimNoEEG selected songs using Model 1 without retraining. Finally, in the “Playlist evaluation” phase, participants pressed a key during chills and rated each playlist on subjective measures related to emotion and well-being (see Table 1 for details).

To clarify the structure of our system, we provide an overview of the main phases of C-BMI: “Recording,” “Modeling,” “Generating playlists,” and “Playlist evaluation” (Figure 1). Additional methodological details of each phase are described in the STAR Methods section.

#### Recording

We defined music chills for participants as a prominent physiological marker, such as “goosebumps” or a “shiver down the spine,” that accompanies intense musical pleasure, to ensure consistency in subjective interpretation across individuals. Importantly, this definition was provided to help standardize how participants evaluated their own experiences, and the chill reports were based entirely on self-reports rather than objective physiological measurements.

We obtained chill-inducing songs from each participant before the experiment and measured their in-ear EEG data while they listened to the songs. Twenty-four participants listened to three songs that had previously induced chills and three additional songs selected by another participant. Each song was played for 90 s. Participants pressed a key whenever they experienced chills while listening to each song and rated their pleasure levels using a visual analog scale (VAS: 0–100) at the end of listening. In-ear EEG data were recorded throughout each song presentation.

#### Modeling

**Figure 2:**
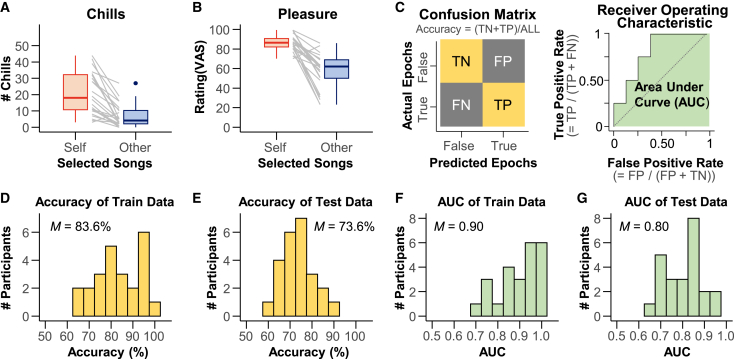
Validation index for the pleasure-predicting model from in-ear EEG (Model 2) (A and B) Number of chills and pleasure ratings for self-selected and other-selected songs in the first “Recording” section. Except for two participants, the participants experienced three or more chills or higher pleasure with self-selected songs than with other-selected songs. Data are represented as boxplots, where the central line indicates the median, the box shows the interquartile range (IQR), and the whiskers extend to the minimum and maximum values within 1.5 × IQR. (C) Schematic drawings of the confusion matrix (left) and receiver operating characteristic (ROC) curve (right). True and false for the predicted and actual EEG epochs refer to the epochs of self-selected and other-selected songs, respectively. The true positive (TP), true negative (TN), false negative (FN), and false positive (FP) values were calculated. The area under the curve (AUC) was calculated using the ROC curve. (D and E) Accuracy of the training and test data. The mean accuracies were 83.6% and 73.6%, respectively. (F and G) AUC of the training and test data. The mean AUCs were 0.90 and 0.80, respectively. Data were represented as histograms. We also confirmed the statistical significance of Model 2 for each participant using a permutation test that shuffled the true and false labels (all *p* < 0.05). For details, refer to the explanation of Model 2 in the STAR Methods section.

We compared the number of chills and pleasure ratings presented in the Recording section: three self-selected by each participant and three selected by the other participant (Figures 2A and 2B). Self-selected songs elicited more chills (20.7 ± 12.9; mean ± SD) and higher pleasure ratings (86.1 ± 8.1) than other-selected songs did (chills: 6.7 ± 7.0, pleasure: 57.1 ± 17.9), except for two participants who were excluded from playlist generation and evaluation analysis. For each participant, we analyzed the acoustic features of the six songs using VGGish,^45^^,^^46^ a pretrained neural network, and developed a linear LASSO model^47^ to predict subjective pleasure levels from the acoustic features (Model 1). Additionally, we created a generalized LASSO model to classify EEG states associated with high pleasure (while listening to self-selected songs) and low pleasure (while listening to other-selected songs) (Model 2). This model was trained on 90% of the data and evaluated on the remaining 10% to ensure that the classification performance was generalized to unseen data. EEG epochs corresponding to self-selected and other-selected songs were labeled as “true” and “false,” respectively. Based on these labels, we constructed a confusion matrix to calculate the classification accuracy and plotted a receiver operating characteristic (ROC) curve to compute the area under the curve (AUC; Figure 2C). Across the participants, the mean training and test accuracies were 83.6% and 73.6%, respectively (Figures 2D and 2E), and the mean AUCs were 0.90 and 0.80, respectively (Figures 2F and 2G). These results indicate that Model 2 can capture neural signals associated with music-induced pleasure.

#### Generating playlists

We generated four tailor-made music playlists for each participant to augment or diminish pleasure, with or without EEG. The playlists were named (1) “augmenting playlist with EEG (AugEEG),” (2) “augmenting playlist without EEG (AugNoEEG),” (3) “diminishing playlist with EEG (DimEEG),” and (4) “diminishing playlist without EEG (DimNoEEG).” We computed the acoustic features of 7,225 candidate songs drawn from the GfK Japan weekly music charts (top 1–1,000, from 2018 to 2022) using VGGish and sorted them based on the pleasure scores predicted by Model 1 and their acoustic similarity to self-selected songs to create a ranking of songs that are likely to induce pleasure.

For Playlists (1) and (2), songs were randomly chosen from the top ranking, whereas for Playlists (3) and (4), songs were randomly chosen from the bottom ranking. The first song in each playlist was randomly selected from the middle range of the ranking (positions 3,577–3,649) to serve as a baseline for analysis. Each playlist comprised seven songs, and each song was played for 90 s. The in-ear EEG was recorded while the participants listened to songs. For the playlists with EEG updates (1 and 3), the decoded pleasure from the EEG using Model 2 and the acoustic features of the song using VGGish were used to retrain Model 1 and update the song ranking. The playlists without EEG updates (2 and 4) did not retrain Model 1 or update the rankings. The participants listened to the four playlists in random order, and the songs in each playlist were uniquely selected using Models 1 and 2, which were created for each participant. Thus, the song order was individualized.

#### Playlist evaluation

The participants pressed a key when they experienced chills while listening to the songs. After completing each playlist, the participants rated 14 items on a VAS (0–100) covering two categories: primary emotion measures (pleasure, excitement, absorption, arousal, valence, preference, and strangeness) and exploratory well-being measures (life purpose, self-acceptance, improving relations, reducing stress, resilience, emotion regulation, and seeking help) (Table 1).

**Table 1:** Subjective rating items

Label	Item
Pleasure	I felt pleasure in the whole playlist.
Excitement	I felt excited throughout the playlist.
Absorption	I became absorbed while listening to this playlist.
Arousal	I am currently aroused.
Valence	I am currently in a comfortable state.
Preference	I prefer this playlist.
Strangeness	I feel strange about this playlist.
Life purpose	Listening to this playlist helped me feel more connected to the purpose and meaning of life.
Self-acceptance	Listening to this playlist helped me accept myself as I am.
Improving relations	Listening to this playlist helped me improve relationships with others.
Reducing stress	Listening to this playlist helped me feel less stressed and more relaxed.
Resilience	Listening to this playlist helped me think about dealing more effectively with difficult situations.
Emotion regulation	Listening to this playlist helped me better understand and accept my emotions and feelings.
Seeking help	Listening to this playlist helped me think about finding support and solutions to the problems and difficulties I am facing.

### Chill counts

**Figure 3:**
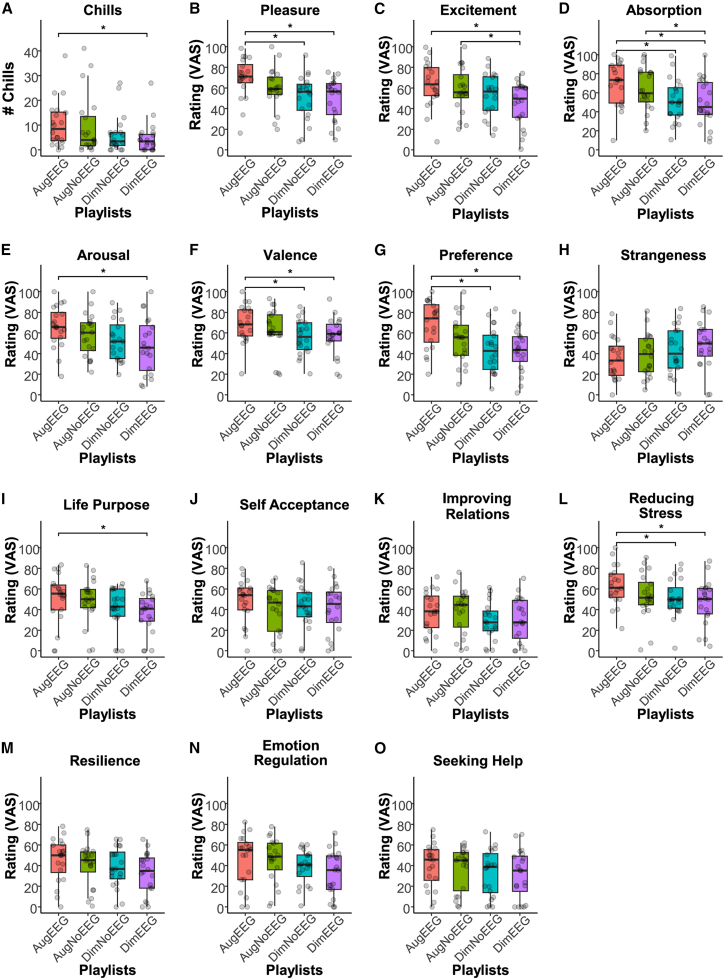
The number of chills and subjective ratings (*n* = 20) Data are represented as boxplots, where the central line indicates the median, the box shows the IQR, and the whiskers extend to the minimum and maximum values within 1.5 × IQR. Each dot represents the value for each participant. (A) Number of chills (# chills) for each playlist. The augmenting playlist with EEG (AugEEG) elicited a significantly larger number of chills than the diminishing playlist with EEG (DimEEG). (B) Subjective pleasure ratings. AugEEG received significantly higher pleasure ratings than the diminishing playlist without EEG (DimNoEEG) and DimEEG. Note that AugNoEEG means the augmenting playlist without EEG. (C–O) Subjective ratings of excitement, absorption, arousal, valence, preference, strangeness, life purpose, self-acceptance, improving relations, reducing stress, resilience, emotion regulation, and seeking help. Subjective ratings were assessed using a VAS. The *p*-values were adjusted using Holm’s method. Asterisks (∗) indicate significant differences (*p*_Holm_ < 0.05, paired Wilcoxon signed-rank tests or paired *t*-tests). See Table 1 for details on the rating items.

To examine whether the number of chills differed across playlists (Songs 2 through 7, excluding the baseline of the first song), we calculated descriptive statistics (mean ± SD) for each playlist and tested for significant differences using nonparametric analyses. Chill counts for the four playlists (Figure 3A) were as follows: AugEEG had the highest count (10.50 ± 9.52; Mean ± SD), followed by AugNoEEG (9.50 ± 12.15), DimNoEEG (6.25 ± 7.64), and DimEEG (4.90 ± 6.37). The normality of the chill counts for all playlists was not confirmed (Table 2). The Friedman test revealed a significant difference in the number of chills across playlists (*χ*^*2*^ = 12.81, *p* = 0.005, *η*^*2*^ = 0.21). Paired Wilcoxon signed-rank tests revealed that the chills of AugEEG were significantly more than DimEEG (*V* = 179.5, *p*_*Holm*_ < 0.001). However, no significant differences were observed between AugEEG and DimNoEEG (*V* = 156.5, *p*_*Holm*_ = 0.055) or between AugEEG and AugNoEEG (*V* = 138.5, *p*_*Holm*_ = 0.242). Additionally, AugNoEEG did not show a significant difference compared to DimEEG (*V* = 128.5, *p*_Holm_ = 0.055) or DimNoEEG (*V* = 123.0, *p*_*Holm*_ = 0.542). DimNoEEG was not significantly different from DimEEG (*V* = 68.5, *p*_*Holm*_ = 0.656). These results suggest that AugEEG induces more chills than DimEEG.

**Table 2:** Descriptive statistics (mean ± SD) for the rating values of each item and chill counts, including results of the Shapiro-Wilk test

Item	AugEEG (*W*-value, *p*-value)	AugNoEEG (*W*-value, *p*-value)	DimNoEEG (*W*-value, *p*-value)	DimEEG (*W*-value, *p*-value)
Chills	**10.50** ± **9.52** **(0.87, 0.013)**	**9.50** ± **12.15** **(0.75, <0.001)**	**6.25** ± **7.64** **(0.75, <0.001)**	**4.90** ± **6.37** **(0.73, <0.001)**
Pleasure	69.47 ± 20.04 (0.92, 0.085)	60.05 ± 19.60 (0.94, 0.285)	51.06 ± 21.90 (0.96, 0.472)	49.17 ± 20.05 (0.913, 0.075)
Excitement	63.27 ± 22.32 (0.97, 0.791)	58.28 ± 21.61 (0.96, 0.600)	52.85 ± 21.00 (0.97, 0.718)	45.24 ± 21.25 (0.92, 0.081)
Absorption	69.42 ± 23.34 (0.93, 0.141)	63.47 ± 22.88 (0.96, 0.637)	53.16 ± 22.76 (0.98, 0.945)	50.11 ± 24.38 (0.97, 0.753)
Arousal	66.05 ± 20.32 (0.97, 0.783)	58.07 ± 20.58 (0.98, 0.899)	52.79 ± 19.81 (0.96, 0.556)	47.71 ± 27.15 (0.96, 0.553)
Valence	68.75 ± 18.30 (0.96, 0.504)	**62.34** ± **21.13** **(0.88, 0.016)**	56.18 ± 17.16 (0.98, 0.972)	56.61 ± 18.73 (0.94, 0.282)
Preference	68.24 ± 22.95 (0.94, 0.244)	53.37 ± 23.30 (0.99, 0.984)	43.44 ± 21.67 (0.97, 0.665)	42.79 ± 20.33 (0.98, 0.959)
Strangeness	35.36 ± 20.67 (0.96, 0.624)	38.46 ± 20.83 (0.97, 0.728)	42.80 ± 22.95 (0.97, 0.832)	48.52 ± 23.42 (0.95, 0.390)
Life Purpose	**50.93** ± **24.25** **(0.90, 0.039)**	48.47 ± 21.84 (0.92, 0.080)	**41.33** ± **20.58** **(0.85, 0.005)**	36.65 ± 19.77 (0.93, 0.127)
Self-acceptance	48.21 ± 20.71 (0.94, 0.202)	40.14 ± 22.73 (0.91, 0.058)	43.09 ± 21.13 (0.98, 0.859)	41.18 ± 23.49 (0.95, 0.439)
Improving relations	37.63 ± 20.10 (0.97, 0.729)	38.98 ± 21.80 (0.93, 0.167)	28.93 ± 18.49 (0.96, 0.522)	29.93 ± 21.48 (0.95, 0.312)
Reducing stress	63.08 ± 19.10 (0.99, 0.995)	53.25 ± 22.65 (0.94, 0.215)	50.86 ± 18.63 (0.93, 0.176)	47.10 ± 21.88 (0.95, 0.424)
Resilience	45.38 ± 21.08 (0.95, 0.341)	**40.83** ± **20.91** **(0.90, 0.047)**	38.27 ± 19.60 (0.95, 0.38)	32.72 ± 20.11 (0.94, 0.281)
Emotion regulation	45.77 ± 25.26 (0.91, 0.069)	45.39 ± 21.75 (0.93, 0.179)	38.59 ± 16.62 (0.93, 0.184)	32.73 ± 21.81 (0.95, 0.327)
Seeking help	40.19 ± 21.21 (0.96, 0.542)	**36.90** ± **21.00** **(0.84, 0.004)**	33.12 ± 22.48 (0.93, 0.175)	31.74 ± 23.45 (0.92, 0.096)

**Note.** Data that did not meet normality assumptions (*p* < 0.05) based on the Shapiro-Wilk test are shown in bold.

### Subjective pleasure rating

To assess whether subjective pleasure ratings (VAS; 0–100), based on the statement “I felt pleasure in the whole playlist” (Table 1), differed across playlists, we summarized the data using descriptive statistics (mean ± SD) and tested for significant differences. The subjective pleasure ratings for the four playlists (Figure 3B) were as follows: AugEEG had the highest rating (69.47 ± 20.04), followed by AugNoEEG (60.05 ± 19.60), DimNoEEG (51.06 ± 21.90), and DimEEG (49.17 ± 20.05). We confirmed the normality of the ratings for each playlist (Table 2). A one-way repeated-measures ANOVA showed that pleasure levels differed significantly across playlists (*F*(3, 57) = 6.31, *p* < 0.001, *η*_*p*_^*2*^ = 0.25, Greenhouse-Geisser (GG) *ε* = 0.82). The paired *t*-tests revealed that the pleasure of AugEEG was significantly higher than that of DimEEG (*t*(19) = 4.17, *p*_Holm_ = 0.003, 95% confidence interval (CI) [10.12, 30.48]) and DimNoEEG (*t*(19) = 3.89, *p*_Holm_ = 0.005, 95%CI [8.50, 28.33]). However, there were no significant differences between AugEEG and AugNoEEG (*t*(19) = 1.51, *p*_Holm_ = 0.443, 95%CI [−3.64, 22.47]). Similarly, AugNoEEG did not show a significant difference compared to DimEEG (*t*(19) = 2.00, *p*_Holm_ = 0.239, 95%CI [−0.50, 22.27]) or DimNoEEG (*t*(19) = 1.47, *p*_Holm_ = 0.443, 95%CI [−3.84, 21.83]). Finally, DimNoEEG was not significantly different from DimEEG (*t*(19) = 0.52, *p*_Holm_ = 0.609, 95%CI [−5.70, 9.48]). These results indicate that AugEEG evokes more pleasure than DimEEG or DimNoEEG.

### Subjective ratings of items related to emotions and well-being

To investigate whether subjective ratings related to emotions and well-being (VAS; 0–100, see Table 1) differed across playlists, we summarized the data using descriptive statistics (see Table 2 for the means and standard deviations of each item) and tested for statistical significance. One-way repeated-measures ANOVAs or Friedman tests revealed significant differences among playlists for the following items (Figures 3C–3O): excitement (*F*(3, 57) = 4.03, *p* = 0.011, *η*_*p*_^*2*^ = 0.17, GG *ε* = 0.78), absorption (*F*(3, 57) = 6.85, *p* < 0.001, *η*_*p*_^*2*^ = 0.27, GG *ε* = 0.86), arousal (*F*(3, 57) = 4.90, *p* = 0.010 (GG corrected), *η*_*p*_^*2*^ = 0.21, GG *ε* = 0.74), valence (*χ*^*2*^ = 14.02, *p* = 0.003, *η*^*2*^ = 0.23), preference (*F*(3, 57) = 9.78, *p* < 0.001, *η*_*p*_^*2*^ = 0.34, GG *ε* = 0.88), life purpose (*χ*^*2*^ = 8.85, *p* = 0.031, *η*^*2*^ = 0.15), and reducing stress (*F*(3, 57) = 3.36, *p* = 0.025, *η*_*p*_^*2*^ = 0.15, GG *ε* = 0.80). Subsequent multiple comparisons (paired *t*-tests or paired Wilcoxon signed-rank tests) revealed that the values of excitement (*t*(19) = 3.01, *p*_*Holm*_ = 0.036), absorption (*t*(19) = 3.57, *p*_*Holm*_ = 0.010), arousal (*t*(19) = 3.04, *p*_*Holm*_ = 0.041), valence (*V* = 179, *p*_*Holm*_ = 0.021), preference (*t*(19) = 5.40, *p*_*Holm*_ < 0.001), life purpose (*V* = 147, *p*_*Holm*_ = 0.034), and reducing stress (*t*(19) = 3.50, *p*_*Holm*_ = 0.012) were higher in AugEEG than in DimEEG. In addition, the ratings of AugEEG were higher than those of DimNoEEG for the following items: absorption (*t*(19) = 3.75, *p*_*Holm*_ = 0.008), valence (*V* = 171, *p*_*Holm*_ = 0.007), preference (*t*(19) = 4.96, *p*_*Holm*_ < 0.001), and reducing stress (*t*(19) = 3.64, *p*_*Holm*_ = 0.011). Excitement (*t*(19) = 3.15, *p*_*Holm*_ = 0.032) and absorption (*t*(19) = 2.88, *p*_*Holm*_ = 0.038) in AugNoEEG were higher than those in DimEEG. However, no significant differences were found among the playlists for the following items: strangeness (*F*(3, 57) = 1.89, *p* = 0.141, *η*_*p*_^*2*^ = 0.09, GG *ε* = 0.83), self-acceptance (*F*(3, 57) = 0.92, *p* = 0.404 (GG corrected), *η*_*p*_^*2*^ = 0.05, GG *ε* = 0.64), improving relations (*F*(3, 57) = 2.35, *p* = 0.082, *η*_*p*_^*2*^ = 0.11, GG *ε* = 0.76), resilience (*χ*^*2*^ = 5.75, *p* = 0.124, *η*^*2*^ = 0.10), emotion regulation (*F*(3, 57) = 2.63, *p* = 0.059, *η*_*p*_^*2*^ = 0.12, GG *ε* = 0.79), and seeking help (*χ*^*2*^ = 3.35, *p* = 0.341, *η*^*2*^ = 0.06). See Tables 3, 4, 5, 6, 7, 8, and 9 for the differences (including the 95% CI) between the playlists.

**Table 3:** Pairwise comparisons for the playlists in the rating of excitement

Contrast	Difference [95%CI Lower, Upper]	*t*-value	df	^*p*^Holm
**AugEEG−DimEEG**	18.03 [5.49, 30.57]	3.01	19	0.036
AugEEG**−**DimNoEEG	10.42 [−0.18, 21.02]	2.06	19	0.215
AugEEG**−**AugNoEEG	4.99 [−9.19, 19.16]	0.74	19	0.605
**AugNoEEG−DimEEG**	13.04 [4.37, 21.72]	3.15	19	0.032
AugNoEEG**−**DimNoEEG	5.43 [−5.30, 16.16]	1.06	19	0.605
DimNoEEG**−**DimEEG	7.61 [−3.25, 18.46]	1.47	19	0.476

**Note.** Contrasts with significant differences from zero (*p*Holm < 0.05) are highlighted in bold. CI represents the confidence interval.

**Table 4:** Pairwise comparisons for the playlists in the rating of absorption

Contrast	Difference [95%CI Lower, Upper]	*t*-value	df	^*p*^Holm
**AugEEG−DimEEG**	19.32 [8.01, 30.63]	3.57	19	0.010
**AugEEG−DimNoEEG**	18.26 [8.07, 28.46]	3.75	19	0.008
AugEEG**−**AugNoEEG	5.95 [−7.25, 19.16]	0.94	19	0.714
**AugNoEEG−DimEEG**	13.36 [3.66, 23.06]	2.88	19	0.038
AugNoEEG**−**DimNoEEG	12.31 [1.87, 22.75]	2.47	19	0.070
DimNoEEG**−**DimEEG	1.05 [−7.70, 9.81]	0.25	19	0.804

**Note.** Contrasts with significant differences from zero (*p*Holm < 0.05) are highlighted in bold. CI represents the confidence interval.

**Table 5:** Pairwise comparisons for the playlists in the rating of arousal

Contrast	Difference [95%CI Lower, Upper]	*t*-value	df	^*p*^Holm
**AugEEG−DimEEG**	18.34 [5.70, 30.98]	3.04	19	0.041
AugEEG**−**DimNoEEG	13.26 [3.21, 23.32]	2.76	19	0.062
AugEEG**−**AugNoEEG	7.98 [0.42, 15.55]	2.21	19	0.159
AugNoEEG−DimEEG	10.36 [−1.58, 22.30]	1.82	19	0.256
AugNoEEG**−**DimNoEEG	5.28 [−5.63, 16.19]	1.01	19	0.496
DimNoEEG**−**DimEEG	5.07 [−3.83, 13.98]	1.19	19	0.496

**Note.** Contrasts with significant differences from zero (*p*_Holm_ < 0.05) are highlighted in bold. CI represents the confidence interval.

**Table 6:** Pairwise comparisons for the playlists in the rating of valence

Contrast	V	^*p*^Holm
**AugEEG−DimEEG**	179	0.021
**AugEEG−DimNoEEG**	171	0.007
AugEEG**−**AugNoEEG	130	0.737
AugNoEEG−DimEEG	156	0.233
AugNoEEG**−**DimNoEEG	146	0.392
DimNoEEG**−**DimEEG	83	0.737

**Note.** Contrasts with significant differences from zero (*p*Holm < 0.05) are highlighted in bold.

**Table 7:** Pairwise comparisons for the playlists in the rating of preference

Contrast	Difference [95%CI Lower, Upper]	*t*-value	df	^*p*^Holm
**AugEEG−DimEEG**	25.45 [15.58, 35.32]	5.40	19	<0.001
**AugEEG−DimNoEEG**	24.80 [14.33, 35.28]	4.96	19	<0.001
AugEEG**−**AugNoEEG	14.87 [1.65, 28.09]	2.35	19	0.118
AugNoEEG−DimEEG	10.58 [−1.38, 22.54]	1.85	19	0.239
AugNoEEG**−**DimNoEEG	9.93 [−2.50, 22.36]	1.67	19	0.239
DimNoEEG**−**DimEEG	0.65 [−8.28, 9.58]	0.15	19	0.881

**Note.** Contrasts with significant differences from zero (*p*_Holm_ < 0.05) are highlighted in bold. CI represents the confidence interval.

**Table 8:** Pairwise comparisons for the playlists in the rating of life purpose

Contrast	V	^*p*^Holm
**AugEEG−DimEEG**	147	0.034
AugEEG−DimNoEEG	142	0.060
AugEEG**−**AugNoEEG	109	0.790
AugNoEEG−DimEEG	127	0.295
AugNoEEG**−**DimNoEEG	101	0.790
DimNoEEG**−**DimEEG	88	0.790

**Note.** Contrasts with significant differences from zero (*p*Holm < 0.05) are highlighted in bold.

**Table 9:** Pairwise comparisons for the playlists in the rating of reducing stress

Contrast	Difference [95%CI Lower, Upper]	*t*-value	df	^*p*^Holm
**AugEEG−DimEEG**	15.98 [6.42, 25.53]	3.50	19	0.012
**AugEEG−DimNoEEG**	12.22 [5.19, 19.25]	3.64	19	0.011
AugEEG**−**AugNoEEG	9.83 [−2.62, 22.28]	1.65	19	0.460
AugNoEEG−DimEEG	6.15 [−6.88, 19.17]	0.99	19	1.000
AugNoEEG**−**DimNoEEG	2.39 [−9.39, 14.17]	0.43	19	1.000
DimNoEEG**−**DimEEG	3.75 [−7.43, 14.94]	0.70	19	1.000

**Note.** Contrasts with significant differences from zero (*p*_Holm_ < 0.05) are highlighted in bold. CI represents the confidence interval.

### Decoded pleasure from in-ear electroencephalogram

**Figure 4:**
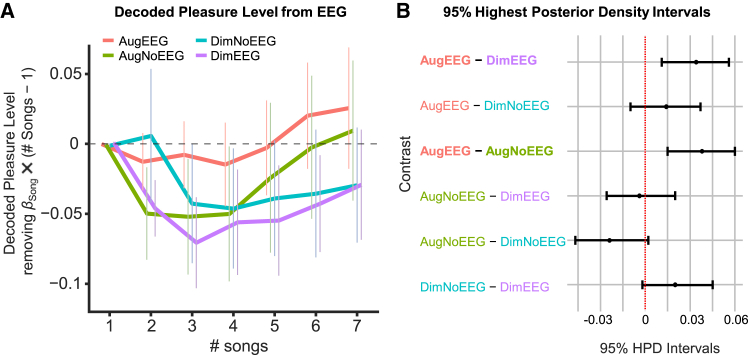
Decoded pleasure levels from in-ear EEG and pairwise comparisons (*n* = 17) (A) Mean decoded pleasure level from in-ear EEG. The Bayesian generalized linear mixed-effects model (GLMM) was applied as follows: *Decoded pleasure ∼ Song (number of songs) + Playlist + (1 | Participants ID)*. The effects of the songs (*β*_Song_ × (*Song* − 1)) were removed from the plot. The pleasure level of the AugEEG increased from the baseline (the first song) as the playlist progressed. Error bars indicate the standard error. (B) 95% highest posterior density intervals of pairwise comparisons. The difference between AugEEG and DimEEG did not include zero, with AugEEG having higher decoded pleasure values than did DimEEG. The difference between AugEEG and AugNoEEG also did not contain zero, with AugEEG having a higher decoded pleasure than AugNoEEG.

To examine whether the pleasure levels decoded from the in-ear EEG differed across playlists, we constructed a Bayesian generalized linear mixed-effects model (GLMM) by putting the baseline-corrected decoded pleasure from the in-ear EEG into the response variable, the number of songs and the playlists into fixed effects, and participant ID into the random effect (Table 10). The baseline was defined as the first 90 s of each playlist, corresponding to the first song that was randomly selected from the middle range of the ranking of the candidate songs (as described in the generating playlists of the Overview of the C-BMI section). The slope of the song (the number of songs) was negative (estimate = −0.009, 95% CI [−0.013, −0.005]), suggesting that as songs progressed in the playlist, the pleasure level decoded from the EEG decreased. After removing the decreasing trend, AugEEG showed the highest decoded pleasure, with an increasing trend from the baseline, followed by AugNoEEG, DimNoEEG, and DimEEG (Figure 4A). Pairwise comparisons between the levels of playlists (Figure 4B; Table 11) revealed a difference between AugEEG and DimEEG (estimate = 0.034, 95% Highest Posterior Density (HPD) intervals [0.011, 0.056]), with AugEEG having higher decoded pleasure values, which is consistent with the results for music chills and the VAS of pleasure. Another difference was found between AugEEG and AugNoEEG (estimate = 0.038, 95% HPD Intervals [0.015, 0.060]), indicating that AugEEG has higher decoded pleasure values than AugNoEEG. The 95% HPD intervals for the other contrasts included zero.

**Table 10:** Estimation of a Bayesian GLMM fitted to decoded pleasure

Variable	Estimated Slope [95%CI Lower, Upper]	SE	R-hat	bulk-ESS	tail-ESS
(Intercept)	−0.034 [−0.077, 0.009]	0.022	1.006	637	1255
**Song**	−0.009 [−0.013, −0.005]	0.002	1.000	8206	3190
**Playlist** **(AugEEG − DimEEG)**	0.034 [0.011, 0.057]	0.012	1.002	2190	2703
Playlist (AugNoEEG − DimEEG)	−0.003 [−0.026, 0.020]	0.012	1.001	2303	2614
Playlist (DimNoEEG − DimEEG)	0.020 [−0.003, 0.044]	0.012	1.001	2327	2447

**Note.** Variables for which the 95% confidence interval (CI) of the estimated slope did not include zero are highlighted in bold. SE stands for standard error. R-hat indicates the potential scale reduction factor, which assesses the convergence of MCMC chains. Bulk-ESS represents the bulk effective sample size, which indicates the number of effectively independent samples in the central part of the posterior distribution. Tail-ESS represents the tail effective sample size, which indicates the number of effectively independent samples in the tails of the posterior distribution.

**Table 11:** Pairwise comparisons for the playlists in decoded pleasure from the EEG

Contrast	Estimate Difference [95% HPD Intervals: Lower, Upper]
**AugEEG−DimEEG**	0.034 [0.011, 0.056]
AugEEG−DimNoEEG	0.014 [−0.010 0.037]
**AugEEG−AugNoEEG**	0.038 [0.015 0.060]
AugNoEEG−DimEEG	−0.004 [−0.026, 0.020]
AugNoEEG−DimNoEEG	−0.024 [−0.047 0.002]
DimNoEEG−DimEEG	0.020 [−0.002, 0.045]

**Note.** The contrasts for which the 95% highest posterior density (HPD) intervals for the estimated difference do not include zero are highlighted in bold.

### Effects of music reward sensitivity and musical sophistication

To examine whether individual differences in music reward sensitivity and musical sophistication influence chills and pleasure derived from the playlists, we collected participants’ scores on the Japanese version of the BMRQ (J-BMRQ),^36^^,^^48^ which includes five subscales (musical seeking, emotional evocation, mood regulation, sensory-motor, and social reward), and the Goldsmiths Musical Sophistication Index (Gold-MSI),^49^^,^^50^ also comprising five subscales (active engagement, perceptual abilities, musical training, singing abilities, and emotions). Furthermore, for each participant, we calculated two difference scores: Chilldiff, defined as the difference in the number of chills between the AugEEG and DimEEG playlists, and Pldiff, defined as the difference in VAS-rated subjective pleasure between the same two playlists. These two indices were then standardized (z-scored) and summed to create a composite “total pleasure EEG score,” reflecting the extent to which the EEG-updated playlist (AugEEG) enhanced pleasure compared to the non-enhancing EEG playlist (DimEEG).

We then performed a stepwise multiple regression analysis with the total pleasure EEG score as the response variable and the ten subscale scores (five from the J-BMRQ and five from the Gold-MSI) as explanatory variables to identify which individual differences in music experience predicted EEG-related enhancement in musical pleasure. A stepwise regression approach was used to determine the final model (*F*(3,13) = 5.73, *p* = 0.010, Adjusted *R*^*2*^ = 0.47; Table 12). The variance inflation factors (VIFs), a measure used to assess multicollinearity among explanatory variables, of all selected variables were less than 2. The normality of the residuals was confirmed (Shapiro-Wilk test: *W* = 0.97, *p* = 0.761). The results suggest that listeners with higher musical training and emotion factor scores on the Gold-MSI experience more pleasure in AugEEG than in DimEEG, whereas higher singing ability scores on the Gold-MSI are associated with less pleasure in AugEEG.

**Table 12:** A stepwise multiple regression model to examine the impact of music reward sensitivity and musical sophistication on overall pleasure with EEG update playlists

Variable	Slope [95%CI Lower, Upper]	SE	*t*-value	*p*-value
(Intercept)	−10.67 [−19.85, −1.49]	4.25	−2.51	0.026
**Musical Training** (Gold-MSI)	0.11 [0.02, 0.21]	0.05	2.49	0.027
**Singing Abilities** (Gold-MSI)	−0.20 [−0.31, −0.09]	0.05	−3.90	0.002
**Emotions** (Gold-MSI)	0.36 [0.13, 0.59]	0.11	3.42	0.005

**Note.** The response variable was “total pleasure EEG,” calculated as follows. We calculated differences in chills (Chilldiff) and subjective pleasure (Pldiff) between the two EEG playlists (AugEEG minus DimEEG). We then calculated the z-scores of the Chilldiff and Pldiff and summed them to obtain the total pleasure score for the EEG playlists (total pleasure EEG). Explanatory variables with *p*-values less than 0.05 are indicated in boldface.

SE stands for standard error.

We also calculated a “total pleasure NoEEG” score to evaluate each participant’s responsiveness to playlists based solely on acoustic features (i.e., without EEG updates). This score was computed by taking the z-scored difference in chill counts (Chilldiff) and pleasure ratings (Pldiff) between the AugNoEEG and DimNoEEG playlists, and summing them. This composite measure reflects how much more (or less) pleasure each participant experienced from an acoustically optimized playlist compared to one designed to diminish pleasure, providing a behavioral index of sensitivity to acoustic-based personalization. A multiple regression model was constructed with the total pleasure NoEEG score as the response variable, a similar stepwise regression approach was utilized, and the final model was determined (*F*(3,13) = 5.18, *p* = 0.014, Adjusted *R*^*2*^ = 0.44; Table 13). The VIFs of all the selected variables were less than 2. The normality of the residuals was confirmed (Shapiro-Wilk test: *W* = 0.98, *p* = 0.983). This result indicates that individuals who may already use music to manage their emotional states derive relatively fewer additional benefits (e.g., chills, enhanced pleasure) from acoustically personalized playlists that do not use EEG-based information.

**Table 13:** A stepwise multiple regression model to examine the impact of music reward sensitivity and musical sophistication on overall pleasure from playlists without EEG updates

Variable	Slope [95%CI Lower, Upper]	SE	*t*-value	*p*-value
(Intercept)	6.14 [−1.15, 13.43]	3.37	1.82	0.092
**Mood Regulations** (BMRQ)	−0.25 [−0.39, −0.10]	0.07	−3.74	0.002
Sensory Motors (BMRQ)	0.05 [−0.04, 0.15]	0.04	1.29	0.219
Perceptual Abilities (Gold-MSI)	0.09 [−0.05, 0.23]	0.07	1.33	0.207

**Note.** The response variable was “total pleasure NoEEG,” calculated as follows. We calculated the differences in chills (Chilldiff) and subjective pleasure (Pldiff) between the two playlists without using the EEG (AugNoEEG minus DimNoEEG). We then calculated the z-scores of Chilldiff and Pldiff and summed them to obtain the total pleasure score (total pleasure NoEEG). Explanatory variables with *p*-values less than 0.05 are indicated in boldface.

SE stands for standard error.

## Discussion

This study aimed to determine whether selecting music based on pleasure decoded from in-ear EEG increased chills and pleasure. AugEEG resulted in the highest number of chills among the playlists, significantly surpassing that of DimEEG (Figure 3A). Similarly, the subjective pleasure ratings were significantly higher for AugEEG than for DimEEG and DimNoEEG (Figure 3B). These findings suggest that personalized song playlists created using C-BMI, a closed-loop neurofeedback system with in-ear EEG, can elicit more chills and greater pleasure. Building on previous studies showing that closed-loop neurofeedback can effectively modulate neural states,^38^ our findings demonstrate that such feedback, using a simpler, portable in-ear EEG system, can enhance chills and pleasure. This suggests that adaptive brain-informed technologies may provide novel ways to enrich emotional experiences through music. Previous studies have used TMS or pharmacological interventions to enhance musical pleasure.^30^^,^^31^ In contrast to these approaches, our method requires no specialized equipment or medical supervision, making it more practical for daily or home use.

Model 2 was used to estimate pleasure levels from the EEG data (Figures 4A and 4B), and we found that the decoded pleasure value for AugEEG exceeded that for AugNoEEG. This result emphasizes that incorporating closed-loop neural feedback enables more effective song selection than preference-based approaches. Indeed, AugEEG consistently outperformed DimEEG across primary outcome measures, including chills, pleasure, excitement, absorption, arousal, valence, and preference^51^^,^^52^ (Figures 3A–3G). In contrast, AugNoEEG, which did not incorporate EEG data, produced significantly higher ratings than DimEEG only for excitement and absorption. Moreover, AugEEG yielded significantly higher ratings than DimNoEEG for pleasure, absorption, valence, and preference, whereas AugNoEEG did not surpass DimNoEEG on any of the primary measures. These findings suggest that AugEEG, which is updated in real time based on EEG activity, consistently enhances emotion-related responses. Current music streaming services, such as Spotify, typically recommend songs based on users’ listening histories,^53^ similar to the approach used for generating AugNoEEG. However, our findings demonstrate that music recommendation systems can move beyond conventional models that rely on acoustic features and user data. By leveraging real-time EEG signals, playlist content can be dynamically adapted to match the listener’s affective state, thereby opening new possibilities for personalized emotion regulation.

A growing body of research has shown that music can promote psychological well-being by improving mood, regulating emotions, and fostering social connections in daily life and clinical contexts.^54^^,^^55^^,^^56^ These benefits have been observed in diverse settings. For example, during the early years of the COVID-19 pandemic, music was widely used as a coping strategy to alleviate feelings of isolation and uncertainty.^57^^,^^58^ In light of this evidence, we included exploratory well-being items based on previous studies,^54^^,^^59^ to assess the potential for future clinical applications. AugEEG elicited higher ratings for life purpose than DimEEG and higher ratings for stress reduction than both DimEEG and DimNoEEG (Figures 3I and 3L). In contrast, AugNoEEG did not yield significant improvements on either measure. These findings suggest that tailoring music using an individual’s EEG signals may enhance well-being more effectively than approaches based solely on acoustic features. Future research could advance brain-music interfaces by incorporating well-being metrics during the model-training phase to further support emotional and psychological health.

In this study, we used the J-BMRQ^36^^,^^48^ and the Japanese version of the Gold-MSI^49^^,^^50^ to assess the effects of participants’ music reward sensitivity and musical sophistication on music chills and pleasure elicited by playlists. The chills and pleasure ratings for AugEEG and DimEEG were converted into z-scores and summed, and a stepwise regression was used to identify which of the 10 factors of music reward sensitivity and music sophistication predicted the total pleasure. The results showed that the slopes of the music training and emotion factors were significantly positive, whereas that of the singing ability factor was significantly negative (Table 12). This indicates that greater music training or higher responsiveness to music emotions correlates with increased pleasure from AugEEG compared to DimEEG. The positive slope of the music-training factor is consistent with recent studies that have reported that music training modulates emotional responses to music.^60^^,^^61^ The emotion factor included questions such as “I sometimes choose music that can trigger shivers down my spine,” which likely explains the amplification of pleasure from AugEEG. Conversely, higher subjective singing ability was associated with greater pleasure from the DimEEG. Individuals with better singing abilities may listen to songs more analytically, making it more difficult to evoke emotions. In a similar stepwise regression model using the difference in total pleasure between AugNoEEG and DimNoEEG as the response variable, mood regulation, sensory-motor, and perception ability were selected as explanatory variables, with only mood regulation being significant and having a negative slope (Table 13). This means that higher emotion regulation is associated with greater pleasure from DimNoEEG. This negative association with mood regulation suggests that individuals who use music to control their emotions may experience fewer benefits from acoustic-based pleasure-inducing playlists. These models also indicate that playlists with EEG updates reflect more individual differences in musical experiences than playlists without EEG updates, supporting the idea that playlists with EEG updates are more personalized in eliciting pleasure.

Chills can be influenced by the structural features of music, such as tempo, key changes, and the presence of vocals.^62^^,^^63^^,^^64^ To assess whether such features may have confounded our main findings, we conducted an additional analysis of all songs used in the four playlist conditions, focusing on 20 participants who provided valid subjective ratings, excluding the first song in each playlist, which served as a baseline (for detailed statistical results, see Table S1). Tempo and key changes were computed using MIRtoolbox (ver. 1.3.4).^65^ A significant difference in tempo was found across playlists, with post-hoc comparisons revealing that AugEEG had a slower average tempo than DimEEG. However, tempo was not significantly correlated with the chill count at the song level. Similarly, no significant differences in key changes were observed across the playlists, and chills were not significantly associated with the number of key changes per song. Regarding vocal presence, over 90% of the tracks in each playlist contained vocals, and there was no significant difference in the chill counts between vocal and non-vocal songs. Taken together, these analyses suggest that the structural characteristics of music, specifically tempo, key changes, and vocal presence, have only a limited influence on chill responses. Nonetheless, future studies should control for musical features to better isolate the unique contribution of EEG-based personalization.

Previous EEG studies have demonstrated that music-induced pleasure and chills are associated with specific neural activities.^39^^,^^40^^,^^41^^,^^42^^,^^43^^,^^44^ For example, Chabin et al.^40^ reported increased theta power in the right prefrontal cortex during music-evoked chills, and Sammler et al.^41^ identified frontal theta activity as a neural correlate of pleasant emotional experiences. Ara and Marco-Pallarés^39^ and Ara et al.^42^ further showed that fronto-temporal theta phase synchronization reflects functional coupling between auditory perceptual and reward-related systems during pleasurable music listening. Additional EEG markers of musical pleasure include frontal alpha asymmetry.^43^ Although these findings highlight specific EEG frequency bands as promising neural markers, they were obtained using multi-channel scalp EEG systems. In contrast, our study employed in-ear EEG with only two electrodes (one in each ear) with limited spatial resolution. Accordingly, we did not limit our analysis to theta or alpha band frequencies. Instead, we adopted a broader frequency range (4–40 Hz) to capture a wider array of potentially informative neural dynamics in our classification model. To assess whether any specific frequency band contributed more than others to the model performance, we computed the average weight values across participants for theta (4–7 Hz; 0.13 ± 0.09), alpha (8–13 Hz; 0.11 ± 0.07), beta (14–30 Hz; 0.11 ± 0.08), and gamma (31–40 Hz; 0.13 ± 0.09) bands. A repeated-measures ANOVA revealed no significant differences in contribution across bands (*F*(1.85, 35.09) = 2.94, *p* = 0.069), suggesting that multiple frequency components contributed comparably to the classifier’s predictions. These weights were derived from the LASSO logistic regression coefficients projected back into the original frequency × electrode space (see STAR Methods for details). Together, these findings support the use of a broadband frequency approach in our in-ear EEG classification pipeline.

A key advancement in this study was the development of a model based on participant-selected songs (Table 14). Many previous studies^15^ have relied on experimenter-selected music. In contrast, our study incorporated songs chosen by each participant, inherently capturing a wider range of musical preferences and reward sensitivities. Using this information, we successfully predicted pleasure from acoustic features and in-ear EEG signals on an individual basis and generated personalized playlists that yielded significant effects. This represents a major strength of the C-BMI.

**Table 14:** Information on self-selected songs and their artists/composers

Song 1	Song 2	Song 3
Melody (Koji Tamaki)	Kaze (Kobukuro)	Towa ni tomoni (Kobukuro)
Neon (John Mayer)	Bad (Christopher)	Finesse (Bruno Mars)
Symphony No.1 (J. Brahms)	Violin Sonata (R. Strauss)	Symphony No.6 (P.Tchaikovsky)
All My Life (Lil Durk)	Imagine (Snoop Dogg)	B2B (BAD HOP)
Love Theme (Ennio Morricone)	Hanataba wo Kimi ni (Hikaru Utada)	Intermezzo Sinfonico (P. Mascagni)
So Sick (Ne-Yo)	Ur Perfect I Hate It, Lenno Remix (Mickey Valen)	Green Green Grass, Sam Feldt remix (George Ezra)
Astrid (glaive)	Gold (Hikaru Utada)	Vivienne Westwood (lileffort)
La traviata Act I (G. Verdi)	This Is Me (Keala Settle)	Locus iste (A. Bruckner)
Flowers feat. Nori (in love with a ghost)	Repeatedly - Video Edit (Ametsub)	Florence (Kevin Penkin)
One Last Kiss (Hikaru Utada)	Kiss & Cry (Hikaru Utada)	Traveling (Hikaru Utada)
Planetarium (Justin Hurwitz)	Hannah Song (Sam Wilkes)	Reve Rouge (Cirque du Soleil)
Piano Concerto in G major (M. Ravel)	McDonald Romance (King Gnu)	Out of This World (Masato Kumoi)
Whenever I GO (Jacob Collier)	Witness Me (Jacob Collier)	Burn The Witch (Radiohead)
Geek U.S.A. (Smashing Pumpkins)	Readymade (Red Hot Chili Peppers)	My Hero (Foo Fighters)
Rose (KID FRESINO)	Numb (Linkin Park)	Dancing Boy (OKAMOTO’S)
Reimei, (Δ)	First Rain (Kontinuum)	Saikai (Seigen Tokuzawa)
Utsukushii hi (SUPER BEAVER)	Symphony No.5 (P. Tchaikovsky)	Seikai (RADWIMPS)
Zakir (John McLaughlin)	Black is the color of my true love’s hair (Nina Simone)	Ex-Factor (Lianne La Havas)
youth ft. Hakushi Hasegawa (KID FRESINO)	Yumeutsutsu (Kenshi Yonezu)	Pale Blue (Kenshi Yonezu)
Ode to Joy (L. Beethoven)	Sonate für Klavier Nr.17 D-Dur D 850 Op.53 (F. Schubert)	FAMILIA (MILLENNIUM PARADE)
Snow White (Keisuke Kuwata)	So Close (Jon McLaughlin)	Part of Your World (Halle Bailey)
The others (MIYAVI)	Uminari (Kodo)	Zoku (Kodo)
Synthesis (tn-shi)	Golden Hour (Soflynaily)	STYX HELIX (MYTH & ROID)
We Are (ONE OK ROCK)	Tremolo (RADWIMPS)	Make A Wish (ELLEGARDEN)

**Note.** Each row shows the self-selected songs provided by each participant. There were no duplicates of the songs among the participants.

The aim of our decoding model was not to detect the precise timing of chill experiences but rather to distinguish between two sustained affective brain states: a high-pleasure state elicited by listening to self-selected music and a low-pleasure state elicited by listening to other-selected music. EEG data for the high-pleasure class were derived from 90-s segments of self-selected songs that were confirmed to include chills within the window, based on real-time button presses and high subjective pleasure ratings. In contrast, the low-pleasure class consisted of matched-length segments from other-selected songs that elicited minimal chills and lower pleasure ratings. While the timing of the chills may have varied within each segment, our model could robustly classify neural features of pleasure, as evidenced by the significant classification performance across participants (Figures 2D–2G).

In conclusion, this study demonstrated that music chills and pleasure can be amplified by estimating pleasure from an individual’s EEG signals and creating a personalized music playlist based on these data. Personalized music experiences of this kind may not only deepen our understanding of the mechanisms underlying musical rewards but also promote listeners’ emotional and psychological well-being. We believe that C-BMI represents a promising step toward more personalized and enriching musical experiences grounded in neuroscience and music research.

### Limitations of the study

This study had several limitations. First, we did not include autonomic physiological measures, such as skin conductance or piloerection, during playlist listening. At this initial stage of C-BMI development, we prioritized subjective evaluations of chills and pleasure to ensure a minimally invasive and naturalistic listening experience that is suitable for both clinical and everyday contexts. To maintain participant comfort and ecological validity, we intentionally avoided attaching external sensors that could interfere with the emotional immersion. Nevertheless, we incorporated an objective neural index of pleasure by decoding it from in-ear EEG signals (Figures 4A and 4B). Notably, the decoded pleasure values were significantly higher during AugEEG than during AugNoEEG. Therefore, we consider EEG-based decoding to be a valid measure of affective states. In addition, participants were instructed to press a button whenever they experienced chills, which we defined as “a prominent physiological marker, such as goosebumps or a shiver down the spine, that accompanies intense musical pleasure.” Accordingly, we interpret the chill reports as a subjective measure that is nonetheless closely grounded in a well-defined physiological phenomenon. To further strengthen these findings, future studies should incorporate objective physiological markers, such as goosebumps, heart rate, and pupil dilation.

Second, because we employed a two-channel in-ear EEG, source localization was not feasible. As a result, we could not determine whether the EEG signals originated from the nucleus accumbens or from other reward-related brain regions. Previous studies have demonstrated that it is possible to estimate nucleus accumbens activity during music listening by combining EEG with functional magnetic resonance imaging.^66^ Therefore, future studies should further elucidate the neural sources of in-ear EEG signals by incorporating multimodal neuroimaging approaches, such as simultaneous EEG-fMRI, to overcome the spatial resolution limitations of EEG and precisely localize activity in reward-related regions.

Third, the GLMM fitted to the decoded pleasure levels indicated a significantly negative slope for the number of songs in the playlist. This suggests that, as the number of songs increased, the overall decoded pleasure level decreased. Considering that it took 40 min to listen to all the playlists, with each song lasting 90 s, it is possible that fatigue from the long measurement and changes in ear conditions affected the decoded pleasure values. Future studies should clarify this decreasing trend and measure pleasure after eliminating these effects.

Finally, in AugEEG and DimEEG, Model 1 was retrained using the pleasure estimation from the EEG after each song. In future studies, it may be possible to update the model using other indices such as subjective ratings or autonomic nervous system activity. Continuous subjective ratings and autonomic nervous system activity, such as heart rate, are promising candidate indices for future model updates. Real-time affective rating paradigms have been successfully used to capture the temporal dynamics of emotional responses to music,^19^^,^^63^^,^^67^ while heart rate has been shown to reflect emotional arousal and is closely associated with music chills.^17^^,^^19^ Future research should explore and compare alternative methods for updating models using EEG data.

## Resource availability

### Lead contact

Further information and requests for resources should be directed to and will be fulfilled by the Lead Contact, Shinya Fujii (fujii.shinya@keio.jp).

### Materials availability

This study did not generate new unique materials.

### Data and code availability

• The data supporting the findings of this study are available from the Open Science Framework repository at https://osf.io/kx7eq/.
• The custom code used in this study is available from the corresponding author upon request.
• Any additional information required to reanalyze the data reported in this article is available from the lead contact upon request.

## Acknowledgments

The authors would like to acknowledge Dr. Ernest Mas-Herrero for his valuable comments on our preliminary results and Mr. Tomohiro Kusutomi for creating the sophisticated graphical abstract. This work was supported by JST 10.13039/501100020953COI-NEXT Grant No. JPMJPF2203 to S.F., and JSPS KAKENHI Grant No. 24KJ1930 to S.K. The funders had no role in the study design, data collection and analysis, decision to publish, or manuscript preparation.

## Author contributions

Conceptualization: S.K., T.E., and S.F. Data curation: S.K. Formal analysis: S.K. Funding acquisition: S.K. and S.F. Investigation: S.K. Methodology: S.K., T.E., Y.S., T.I., and S.F. Project administration: S.F. Software: S.K. and T.I. Supervision: S.F. Visualization: S.K. Writing – original draft preparation: S.K. Writing – review and editing: S.K., T.E., Y.S., Y.N., Y.I., T.I., and S.F.

## Declaration of interests

The authors have read the journal’s policy and have the following competing interests: All authors associated with this research are employed by VIE, Inc. This does not alter our adherence to the journal’s policy of sharing data and materials.

## STAR★Methods

### Key resources table

**Table:** 

REAGENT or RESOURCE	SOURCE	IDENTIFIER
**Deposited data**		

Data	OSF	https://osf.io/kx7eq/

**Software and algorithms**		

MATLAB R2022a	https://www.mathworks.com/products/matlab.html	RRID: SCR_001622
R (ver 4.1.1)	https://www.r-project.org/	RRID: SCR_001905
G∗Power (ver 3.1.9.7)	https://www.psychologie.hhu.de/arbeitsgruppen/allgemeine-psychologie-und-arbeitspsychologie/gpower	RRID: SCR_013726

### Experimental model and study participant detail

We recruited twenty-four participants (10 males, 14 females; 23.8 ± 6.5 years old) and obtained written informed consent from all participants. All experimental procedures were approved by the Research Ethics Committee of Keio University Shonan Fujisawa Campus, Japan (#524). The experiment was conducted from February 14, 2024, to May 3, 2024. The sample size of 24 participants was determined beforehand using G∗Power (ver. 3.1.9.7) software for one-way repeated-measures analysis of variance (ANOVA), assuming a medium-sized effect, *α* = 0.05, and *power* = 0.8.^68^

The effect of participant sex on the primary outcomes (Figure 3) was not a central objective of this study and was therefore not analyzed, as the valid sample consisted of an unequal number of males and females (7 males, 13 females). To address this limitation, future studies should recruit larger and more balanced samples to allow for appropriate analysis of sex-related effects.

### Method details

An overview of this method is shown in Figure 1. The experiment was conducted using MATLAB (2022a; Mathworks, USA).

#### Recording

##### Self-selected and other-selected songs

Participants were informed that music chills are “a prominent physiological marker such as “goosebumps” or a “shiver down the spine” that accompany intense musical pleasure” and were asked whether they had experienced the sensations. To reduce variability in interpretation, we provided this definition as a reference for participants, noting that chills were measured through self-report without the use of physiological sensors in the experiment. After confirming the prior experiences, each participant selected three songs (including instrumentals) during which they personally experienced music chills multiple times. The participants provided songs as YouTube links or mp3 files to the first author (the experimenter) before the experiment day. Previous research indicates that pleasure can be triggered by specific musical events.^21^ Thus, we requested that the participants specify the moments within the songs when they were most likely to experience chills. See Table 14 for the song information.

##### In-ear EEG with the number of chills and pleasure ratings

Participants listened to three self-selected songs and three songs selected by another participant in a random order. The assignment of songs selected by another participant was random, with no duplicates between participants. The playback time for each song was 90 s, which included the parts where the participants frequently experienced chills. Determining the playback segment based on the timing of chills reported prior to the experiment is broadly consistent with previous research that aligned physiological measurements with emotionally salient moments, such as the chorus entrance.^21^ If no specific time was reported, the first 90 s of the song was played. The participants adjusted their volume to a comfortable level before the playback.

While listening to the songs, we measured the participants’ in-ear EEG using the VIE CHILL device (VIE Inc., Japan). EEG data were recorded from the left and right ear canals with a reference electrode placed on the back of the neck. We attached electrically conductive ear tips to two electrodes inserted into the ear canals. These electrodes also functioned as earphones, allowing simultaneous song playback and EEG collection. The sampling rate was set at 600 Hz. The participants pressed the C key on the keyboard when they experienced chills. To avoid noise from other physical movements, such as electromyography, the participants were instructed to remain relaxed and avoid moving, except when they pressed the key. At the end of each song, the participants rated their level of pleasure on a visual analog scale (VAS) from 0 to 100. After the evaluation, the next song was played, and the EEG recording was restarted.

#### Modeling

##### Model 1: A pleasure predicting model from acoustic features

For each participant, the acoustic features of the six 90-second song excerpts (three self-selected and three other-selected) were calculated using VGGish,^45^^,^^46^ a pretrained deep convolutional neural network developed by Google. Specifically, VGGish transforms raw audio waveforms into 128-dimensional embedding vectors for each timeframe, thereby capturing low- to mid-level spectrotemporal characteristics. In this study, we used these embeddings to predict participants’ subjective pleasure ratings. VGGish extracted 372 time frames (determined based on 90-second duration) and 128 feature dimensions. The values of the acoustic features were averaged for each time frame corresponding to 10 s, resulting in a matrix of 6 songs × 9 means × 128 dimensions.

We calculated the mean and standard deviation of the features across the six excerpts for each dimension and computed the z-scores of the acoustic features. Additionally, we adjusted the z-scores by selecting dimensions where the mean was not a missing value (NaN) and the standard deviation was greater than 0.01, to improve the accuracy of the model. Finally, we constructed a linear LASSO model^47^ with the z-scores of the pleasure ratings as the response variable and the adjusted z-scores of the acoustic features as the explanatory variables as

(Equation 1) $$\hat{y_{i}}=\hat{\beta_{0}}+{\hat{\beta }}^{T}x_{i}$$

where $\hat{y_{i}}$ is the predicted subjective pleasure rating for song *i*. $\hat{\beta_{0}}$ is the intercept, and $\hat{\beta }$ is a vector of coefficients for each acoustic feature dimension. ***x***_***i***_ is a vector of selected acoustic feature dimensions calculated by VGGish for song *i*. Note that $\hat{\beta_{0}}$ and $\hat{\beta }$ are the variables of *β*_0_ and ***β*** that minimize the following loss function:

(Equation 2) $$\min_{\beta_{0},\beta }\left\{\frac{1}{N}\sum_{i=1}^{N}{\left(y_{i}-\left(\beta_{0}+\beta x_{i}\right)\right)}^{2}+\lambda \left|\left|\beta \right|\right|\right\}$$

where λ is the regularization parameter. The optimal λ was selected using 5-fold cross-validation. LASSO applies an L1 penalty that shrinks less informative coefficients toward zero, resulting in a sparse model with reduced variance and a lower risk of overfitting. *N* represents 6, the number of songs.

##### Model 2: A pleasure predicting model from in-ear EEG

To develop a model for classifying EEG signals into states of high or low pleasure, we tested whether participants experienced more chills and a higher level of pleasure with the songs they chose themselves than with songs chosen by another participant (Figures 2A and 2B). The criterion for chills was that they experienced three or more chills in self-selected songs compared to other-selected songs, based on previous research.^14^ Furthermore, the criterion for pleasure was that the average level of the three self-selected songs should be higher than that of the songs chosen by others. Two participants did not meet the inclusion criteria.

For preprocessing the EEG data, the raw data were filtered using a fourth-order Butterworth filter with a frequency range of 3–40 Hz. We then performed an independent component analysis (ICA) using the filtered signals from the left and right ear electrodes and extracted two components.^69^^,^^70^ The root mean square (RMS) of each component was calculated, and the component with the largest RMS, which was presumed to contain noise (e.g., muscle or movement-related artifacts), was identified and removed from the data. All subsequent analyses were performed on the reconstructed sensor-space signal, not on the ICA component space. This RMS-based criterion for component removal has been validated in previous research using in-ear EEG for music-evoked emotional state decoding.^71^^,^^72^ A sliding window of 4 s (with a 50% overlap) was then applied to the continuous EEG recordings within each song to extract epochs. We calculated the power spectral density of these epochs from 4 to 40 Hz at 0.5 Hz intervals and the sum of the powers across all frequency bands to obtain the RMS for the two channels (left and right ears). In addition, we obtained the maximum value of the numerical gradient of the signal and the skewness in the same manner. Each noise metric was standardized and averaged for the right and left electrodes and each time window, and a noise flag was set for data that exceeded the predetermined threshold of 2.5. This noise rejection threshold is consistent with previous studies that used a similar in-ear EEG device.^71^^,^^72^ These preprocessing procedures were implemented to minimize artifact contamination while preserving neural signals. After artifact rejection, the remaining epochs were used for the classification. Across the participants, the average number of non-flagged epochs was 193.2 (*SD* = 48.6).

The EEG data were then standardized for all non-flagged epochs, frequency windows, and channels (left, right, and their difference). We calculated the power spectral features extracted from each epoch and constructed a feature matrix in which each row represents a single epoch and each column corresponds to the power value for a specific frequency bin and channel type. Power values were computed from 4 to 40 Hz in 0.5 Hz steps (yielding 73 frequency bins) across the three spatial channels, resulting in 219 features per epoch (73 × 3). To reduce the dimensionality of the data, principal component analysis (PCA) was performed separately for each participant without pooling the data across the participants. We retained up to 150 principal components per participant to maintain sufficient information and to avoid overfitting. If a participant had fewer than 150 epochs, all the available components were retained. This upper limit was determined based on previous studies that utilized in-ear EEG classification.^71^^,^^72^^,^^73^ The mean and standard deviation of the principal component scores were calculated and standardized. Each epoch was labeled based on whether it was recorded while the participant listened to self-selected (“true”) or other-selected (“false”) songs. When the numbers of true and false epochs were imbalanced, the epochs were randomly subsampled from the larger group to match the size of the smaller group.

After class balancing, each participant’s data were randomly split into a training set (90%) and a test set (10%). Within the training set, we performed a 20-fold cross-validation to determine the optimal value of the regularization parameter for the logistic LASSO regression model, which was used to decode pleasure from the EEG frequency components. The test set, which was never used during model fitting or hyperparameter tuning, served as an independent holdout dataset for evaluating the out-of-sample classification performance. This two-stage procedure ensures (i) the prevention of overfitting during parameter tuning and (ii) the assessment of generalization to the unseen data. The model’s prediction for the odds of pleasure, denoted as $\hat{Y_{j}}$, is expressed as

(Equation 3a) $$\hat{Y_{j}}=\log\left(\frac{p_{j}}{1-p_{j}}\right)=\hat{\gamma_{0}}+{{\hat{\gamma }}_{j}}^{T}X_{j}$$

(Equation 3b) $$Y_{j}=\left\{ \begin{array}{c} 1\\ 0 \end{array}\right.,P\left(Y_{j}=1\right)=p_{j}$$

where *p*_*j*_ is the probability that the EEG data are classified into a state when listening to self-selected songs, *j* is the number of EEG components, $\hat{\gamma_{0}}$ is the intercept, and $\hat{\gamma }$ is a vector of coefficients corresponding to each EEG component. ***X***_***j***_ represents a vector of the EEG components. Note that *Y*_*j*_ is the binary outcome variable. $\hat{\gamma_{0}}$ and $\hat{\gamma }$ are the variables of *γ*_0_ and ***γ*** that minimize the following loss function:

(Equation 4) $$\min_{\gamma_{0},\gamma }\left\{\frac{1}{M}\sum_{j=1}^{M}{\left(Y_{j}-\left(\gamma_{0}+\gamma X_{j}\right)\right)}^{2}+\lambda \left|\left|\gamma \right|\right|\right\}$$

where *λ* is the regularization parameter. *M* represents the maximum value of the EEG components.

We calculated the confusion matrix, including true positives, false positives, false negatives, and true negatives, and the accuracy rate of the training data, as well as the receiver operating characteristic (ROC) curve and the area under the curve (AUC) (Figure 2C). The ROC curve and the area under the curve (AUC) for the test data were also calculated. The mean accuracy rates for the trained and test data were 83.6% and 73.6%, respectively (Figures 2D and 2E), and the mean AUC for the trained and test data were 0.90 and 0.80, respectively (Figures 2F and 2G). We performed a nonparametric permutation test for each participant to assess the statistical significance of the classifier performance. Binary labels (self-selected vs. other-selected) were randomly shuffled 1,000 times, and the entire classification procedure was repeated using the same 90%/10% training–testing split for every permutation. At each iteration, we computed the test-set AUC, yielding a null distribution under the assumption that there was no relationship between the EEG features and pleasure labels. The observed AUC for each participant was compared to the null distribution. The *p*-value was defined as the proportion of permuted AUCs that exceeded the observed AUC. All participants showed *p*-values below 0.05, confirming that the classifier performance was significant (see the OSF repository in Data and Code Availability for each participant’s result). As analyzed earlier, participants experienced more chills and higher subjective pleasure with the songs they selected than with songs selected by others (Figures 2A and 2B). Hence, this generalized logistic LASSO model that classifies true and false epochs can be used to estimate pleasure from the EEG.

To help control for potential bone conduction artifacts in in-ear EEG recordings, we deliberately employed a within-subject comparison design, classifying EEG responses to self-selected (high pleasure) versus other-selected (low pleasure) songs, both delivered through earphones under acoustically identical conditions. This design choice not only allowed us to isolate neural responses associated with affective engagement, but also helped mitigate bone conduction artifacts, as any acoustic vibrations transmitted through the skull would be present to a similar extent in both conditions. By keeping the acoustic transmission pathways constant, the decoder can focus on differences in neural activity rather than on stimulus-driven physical artifacts. This rationale is consistent with prior studies that used the same in-ear EEG system to decode music-related brain states.^71^^,^^72^

To supplementally examine which frequency bands contributed most to the classification of high- versus low-pleasure music listening, we computed weight maps for each frequency band based on the trained decoding models. We obtained the LASSO logistic regression coefficients for the standardized principal component scores of the EEG power. These coefficients were then rescaled by the corresponding PCA standard deviations and multiplied by the PCA loadings to back-project them into the original frequency × electrode space. This procedure allowed us to visualize the relative contribution of each frequency band (theta: 4-7 Hz, alpha: 8-13 Hz, beta: 14-30 Hz, and gamma: 31-40 Hz) to the model’s predictions.

#### Generating playlists

##### Songs and playlist generation

The candidate songs for the playlists comprised 7,225 tracks from the GfK Japan weekly music chart (www.billboard-japan.com/charts/ ), covering the Top 1,000 entries from April 2018 to February 2022. The feature values of these candidate songs were calculated using VGGish. These values were then normalized, with values exceeding two standard deviations being clipped. A matrix was created to predict subjective pleasure levels using Model 1. The scores were calculated by adding the z-scores of the correlation coefficients of the feature values of each candidate song to the participants’ self-selected songs. The candidate songs were then ranked in descending order based on these scores.

Each participant listened to four playlists, each containing seven songs. Each song lasted 90 s. The four playlists were (1) augmenting playlist with EEG (AugEEG), (2) augmenting playlist without EEG (AugNoEEG), (3) diminishing playlist with EEG (DimEEG), and (4) diminishing playlist without EEG (DimNoEEG). The first song in each playlist, used as a baseline, was randomly selected from positions 3,577 to 3,649 in the candidate-song ranking. AugEEG used the retrained Model 1 and an updated song ranking (see retraining of model 1 using EEG section), selecting songs from the top 72 tracks (top 1% of all songs). In contrast, AugNoEEG used the initial Model 1 without EEG-based retraining; thus, the ranking remained fixed. To avoid overlapping with AugEEG, this playlist selected songs from the top 432 tracks (top 6%). Because AugEEG selected from the top 72 tracks repeatedly across six songs, this also resulted in 432 songs (with possible duplicates). To match this number, the selection range for AugNoEEG was set to 432 songs. Similarly, DimEEG selected songs from the bottom 72 tracks (bottom 1%) based on retrained Model 1, whereas DimNoEEG used the initial Model 1 without EEG-based retraining and selected songs from the bottom 432 pieces (bottom 6%). The participants experienced the four playlist conditions in a random order, and the specific song content and order within each playlist were individualized using Models 1 and 2, which were constructed for each participant.

##### In-ear EEG measurement and decoding

EEG data were measured during playlist listening at a sampling rate of 600 Hz and a frequency range of 4-40 Hz. The EEG data were processed using a subset of the preprocessing steps described in the Modeling section. A Butterworth filter was applied to the signal in the 3-40 Hz band. The filtered EEG data were used to calculate the power spectrum, which was normalized and input into Model 2 to predict the pleasure. For noise determination, metrics such as RMS, maximum gradient, and kurtosis were calculated, and a noise flag was set. ICA-based artifact rejection was omitted at this stage to enable real-time decoding during the experimental session. The moving average of the decoded pleasure state was calculated every 5 s, resulting in one data point per second. If the length of the decoded pleasure data was less than five, the average of the data points that were not flagged as noise was calculated. If it was five or more, the average of the latest five data points that were not flagged as noise was calculated.

##### Retraining of Model 1 using EEG

After each song presentation during the EEG-updated playlist phase (AugEEG and DimEEG), Model 1 was retrained using the newly decoded pleasure estimates from the EEG as the response variable, and the acoustic features of the corresponding song as predictors. $\bar{\hat{Y}}$, the average predicted pleasure for 90 s calculated by Model 2, excluding noise sections, was converted to an approximate value of a VAS, $\hat{y^{\prime }}$, as follows:

(Equation 5) $$\hat{y^{\prime }}=\left(VAS_{\max}-VAS_{\min}\right)*\bar{\hat{Y}}+VAS_{\min}$$

where *VAS*_*max*_ and *VAS*_*min*_ are the maximum and minimum VAS ratings in the first Recording section, respectively. $\hat{y^{\prime }}$ was standardized using the mean and standard deviation of the VAS ratings. Model 2 (the logistic LASSO classifier) outputs a probability-like value between 0 and 1. Scaling this value to the observed minimum and maximum VAS ratings allowed Model 2 to express EEG-derived pleasure estimates in terms directly comparable to the participants’ self-reports. The acoustic features of the song were pre-calculated using VGGish and converted to z-scores. Model 1 was retrained using the original six data points (three self-selected songs and three other-selected songs) and a newly added data point from the playlist song. A linear LASSO regression procedure and hyperparameter selection (λ via 10-fold cross-validation) were applied. This retraining step was repeated after each song in the playlist to dynamically update the song rankings.

#### Playlist evaluation

The participants pressed the C key when they experienced chills while listening to each song. At the end of each playlist, they reflected on the entire playlist and rated it on 14 items (Table 1) using the VAS (0–100). Primary outcome measures other than chills (pleasure, excitement, absorption, arousal, valence, preference, and strangeness) were adapted from previous studies on affective responses to music.^51^^,^^52^ Seven additional exploratory well-being items, including “life purpose,” “self-acceptance,” “improving relations,” “reducing stress,” “resilience,” “emotion regulation,” and “seeking help,” were informed by the literature on music and well-being.^54^^,^^59^ After listening to all playlists, participants completed the Japanese version of the Barcelona Music Reward Questionnaire (J-BMRQ^48^) and the Japanese version of the Goldsmiths Musical Sophistication Index (Gold-MSI^49^).

### Quantification and statistical analysis

All statistical analyses were performed using R software version 4.1.1.^74^ In the analysis of the chill counts and VAS ratings (Figure 3, Table 1), we excluded four participants and analyzed the data from 20 participants. Two participants did not experience three or more chills^14^ or higher pleasure from self-selected songs than from other-selected songs in the previous modeling section (Figures 2A and 2B). Additionally, the pleasure level decoded from the EEG of one participant was consistently 0.9 or higher across the three playlists, indicating a failure in decoding and feedback. The fourth participant was excluded because more than 30% of their EEG data during the DimEEG were judged to be noise. We calculated the number of music chills (excluding the first song of each playlist, which served as a baseline) and the VAS score for pleasure for each playlist (Figures 3A and 3B, Table 2). We hypothesized that AugEEG would induce more chills and higher pleasure than the other playlists (AugNoEEG, DimNoEEG, and DimEEG). To check whether these values differed between playlists, we performed a one-way repeated-measures analysis of variance (ANOVA) for normally distributed data. Subsequent paired t-tests were used to examine the differences between specific playlists. For non-normally distributed data, the Friedman test and subsequent paired Wilcoxon signed-rank test were used. Normality assumptions were checked using the Shapiro-Wilk test. Similar analyses were performed for all other items (Figures 3C–3O, Table 2). The *p*-values for multiple tests were corrected using Holm’s method (denoted as *p*_*Holm,*_ Asterisks indicate *p*_*Holm*_ < 0.05 in Figure 3). As measures of effect size, we calculated partial eta-squared for one-way ANOVAs and eta-squared for Friedman tests.

To examine whether the pleasure levels decoded from the EEG differed among playlists, we constructed a Bayesian generalized linear mixed-effects model (GLMM) (Figure 4A, Table 10) using the ‘brms’ package in R (ver. 2.16.3).^75^^,^^76^^,^^77^ We excluded three additional participants from the analysis because more than 30% of their EEG data were missing during DimNoEEG, resulting in 17 participants. The GLMM was specified using the following formula:

(Equation 6) Decodedpleasure∼Song+Playlist+(1|ParticipantID)

The response variable was the baseline-corrected decoded pleasure from the in-ear EEG (mean per song). The model included fixed effects for Song (number of songs) and Playlist and a random intercept for each participant to account for individual differences. Playlists were treated as categorical factors, with the DimEEG set as the reference level for comparisons. The priors for the model parameters were set to the default non-informative priors provided by brms, except for the intercepts and standard deviations, which were left unspecified. We assumed that the residual errors followed a Student’s t-distribution to accommodate potential outliers and heavier tails in the data. Markov Chain Monte Carlo (MCMC) sampling was performed with the following settings: 4 chains, 2,000 iterations per chain, 1,000 warm-up samples per chain, resulting in 4,000 total post-warm-up samples. The convergence of the MCMC chains was assessed using the potential scale-reduction factor (R-hat). After fitting the GLMM to the data, we conducted pairwise comparisons between playlists using the ‘emmeans’ package (ver. 1.10.3).^78^ We computed the estimated marginal means and performed pairwise contrasts with 95% highest posterior density (HPD) intervals (Figure 4B, Table 11). We evaluated whether the 95% confidence interval of the estimated slope included zero. For subsequent comparisons, we evaluated whether the 95% HPD interval included zero.

Finally, we performed stepwise multiple regression analyses to explore the J-BMRQ and Gold-MSI factors that influence the pleasure of playlists with and without EEG. We conducted the analysis on the same 17 participants as in the previous GLMM. We calculated differences in chills (Chilldiff) and subjective pleasure (Pldiff) between the two EEG playlists (AugEEG minus DimEEG). We then calculated the z-scores of Chilldiff and Pldiff and summed them to obtain the total pleasure score for the EEG playlists (total pleasure EEG). We created a model with 10 factors (five from J-BMRQ and five from Gold-MSI) as explanatory variables, and total pleasure EEG as the response variable. A stepwise regression approach was used to determine the optimal model by searching for the lowest Akaike Information Criterion (AIC) value (Table 12). We also calculated the total pleasure score by subtracting the pleasure score of DimNoEEG from that of AugNoEEG (total pleasure NoEEG: AugNoEEG minus DimNoEEG). Another multiple regression model was constructed with the total pleasure NoEEG score as the response variable using a similar stepwise method (Table 13). We used the ‘stepAIC’ function in the ‘MASS’ package (ver. 7.3.54).^79^ To calculate the variance inflation factor (VIF) for the optimized models, we used the ‘vif’ function from the ‘car’ package (ver. 3.1.2).^80^ All tests were two-sided with a significance level of *α* = 0.05.
