# Supplementary material for: A chill brain-music interface for enhancing music chills with personalized playlists
Source: iScience. 2025 Dec 19;29(1):114508. doi: 10.1016/j.isci.2025.114508 (PMC12809743; doi:10.1016/j.isci.2025.114508)
Supplement: Document S1. Table S1 [file mmc1.pdf]

iScience, Volume *29*

Supplemental information

A chill brain-music interface for enhancing music chills with personalized playlists

Sotaro Kondoh, Takahide Etani, Yuna Sakakibara, Yasushi Naruse, Yasuhiko Imamura, Takuya Ibaraki, and Shinya Fujii

Supplemental information

Table S1. Tempo (bpm: beats per minute, mean ± SD) and number of key changes (mean ± SD) averaged across participants, and total number (and percentage) of vocal tracks included in each playlist (related to Discussion).

	AugEEG	AugNoEEG	DimNoEEG	DimEEG
Tempo	133.68 ± 9.07	131.96 ± 7.95	139.13 ± 11.32	139.78 ± 9.49
Key changes	2.22 ± 1.13	1.60 ± 0.87	1.48 ± 0.61	1.40 ± 0.72
#Vocal tracks (%)	118 (98.3%)	120 (100.0%)	114 (95.0%)	111 (92.5%)

These data were based on 20 participants who provided valid subjective ratings, excluding the first song in each playlist, which served as a baseline. Thus, the total number of songs analyzed in each playlist was 120.

A one-way repeated-measures ANOVA revealed a significant difference in tempo across the playlists ($F(3,57) = 3.16$, $p = 0.031$). Post hoc comparisons indicated that AugEEG had a significantly slower tempo than DimEEG ($t = 2.95$, $df = 19$, $p_{Holm}=0.049$), while other pairwise comparisons were not significant: AugNoEEG vs. DimEEG ($t = 2.53$, $df = 19$, $p_{Holm}=0.102$), AugNoEEG vs. DimNoEEG ($t = 2.47$, $df = 19$, $p_{Holm}=0.102$), AugEEG vs. DimNoEEG ($t = 1.57$, $df = 19$, $p_{Holm}=0.401$), AugEEG vs. AugNoEEG ($t = 0.62$, $df = 19$, $p_{Holm}=1.000$), and DimNoEEG vs. DimEEG ($t = 0.16$, $df = 19$, $p_{Holm}=1.000$). However, tempo did not significantly correlate with the chill counts at the track level ($r_s = -0.07$, $p = 0.111$; Spearman's rank correlation).

A Friedman test revealed no significant differences in the number of key changes across the playlists ($\chi^2 = 4.78$, $df = 3$, $p = 0.189$). Similarly, the number of key changes per track was not significantly correlated with the chill counts ($r_s = 0.08$, $p = 0.088$, Spearman's rank correlation).

Finally, a Wilcoxon rank-sum test revealed no significant difference in the chill counts between vocal tracks (1.32 ± 2.07, 463 tracks) and non-vocal tracks (1.06 ± 1.75, 17 tracks) ($W = 3634$, $p = 0.562$).
